# The complete mitochondrial genome of *Linnemannia amoeboidea* (W. Gams) Vandepol & Bonito (*Mortierellales*: *Mortierellaceae*)

**DOI:** 10.1080/23802359.2022.2039080

**Published:** 2022-02-13

**Authors:** Yang Yang, Xiao-Yong Liu, Bo Huang

**Affiliations:** a Anhui Provincial Key Laboratory for Microbial Pest Control, Anhui Agricultural University, Hefei, China; b College of Life Sciences, Shandong Normal University, Jinan, China

**Keywords:** *Linnemannia*, *Mortierellomycotina*, mitochondrion, phylogenetic analyses

## Abstract

The complete mitochondrial genome of *Linnemannia amoeboidea* (W. Gams) Vandepol & Bonito 2020 (Strain no.: CBS 889.72) was sequenced under the next-generation sequencing platform. It was the second one in the family *Mortierellaceae* Luerss. 1877. The circular genome was 49,702 bp in size, with a GC content of 20.86%. Gene prediction revealed 15 PCGs, two rRNA genes, 26 tRNA genes, one *rnpB* gene and seven ORFs. Phylogenetic analyses showed that *L. amoeboidea* was closely related to *Podila verticillate* (Linnem.) Vandepol & Bonito 2020.

In the newly proposed framework, the species *Mortierella amoeboidea* W. Gams 1976 was reclassified into the genus *Linnemannia* Vandepol & Bonito 2020 as a new combination *L. amoeboidea* (W. Gams) Vandepol & Bonito 2020 (Vandepol et al. 2020). This genus is widely distributed and can be easily isolated from soil, plant debris, insect, etc. (Gams 1977). Some species of *Linnemannia* Vandepol & Bonito 2020 produce polyunsaturated fatty acids, which have potential applications in industrial bioenergy (Zhao et al. 2021). Though as many as 120 species are recognized in *Mortierellaceae* Luerss. 1877, just one has mitogenome available in GenBank (https://www.ncbi.nlm.nih.gov/genome/), which limits the comprehensive and in-depth understanding of this group of fungi. Herein, the mitogenome of *L. amoeboidea* (W. Gams) Vandepol & Bonito 2020 is analyzed and its phylogenetic position is inferred.

The ex-type strain CBS 889.72 of *Linnemannia amoeboidea* (W. Gams) Vandepol & Bonito 2020 was collected from Teutoburger Wald, Beller Holz, Germany (51°9′ N, 8°8′ E) and preserved at Westerdijk Fungal Biodiversity Institute (Utrecht, The Netherlands).

Fungal culture was incubated on PDA for 1 week at 25 °C. Total genomic DNAs were extracted from fresh fungal mycelia using modified CTAB method (Watanabe et al. 2010). The duplicate specimen and genomic DNA was deposited at China General Microbiological Culture Collection Center, Beijing, China (http://www.cgmcc.net/, You-Zhi Wang, yzwang@im.ac.cn) under the voucher number CBS 889.72. By Illumina HiSeq X-ten sequencing (Nextomics Biosciences, Co., Ltd., Wuhan, China), paired-end libraries with 300 bp inserts were constructed according to the manufacturer’s instructions (Biooscientific, AI™ Paired-End DNA Sequencing Kit). We conducted a quality assessment to obtain clean reads from raw sequencing data by FastQC 0.11.8 (Andrews 2010). After that, the mitogenome was assembled from clean data by NOVOPlasty (Dierckxsens et al. 2017) with *Podila verticillata* (Linnem.) Vandepol & Bonito 2020 (NC_006838) as a reference sequence. We annotated the complete mitogenome using the same method as described in previous studies (Zhang et al. 2017; Li et al. 2021). Briefly, the mitogenome annotation was preliminarily conducted by MFannot (http://megasun.bch.umontreal.ca/cgi-bin/mfannot/mfannotInterface.pl) using the mitochondrial genetic code (genetic code 4) to predict mitogenome organization. The transfer-RNA (tRNA) annotations were identified using tRNAscan-SE v1.3.1 (Lowe and Eddy 1997). Intronic and intergenic spacers were searched by ORF Finder (http://www.ncbi.nlm.nih.gov).

The complete mitogenome sequence of *Linnemannia amoeboidea* (W. Gams) Vandepol & Bonito 2020 was deposited in GenBank under the accession number of MZ411570. It is circular and 49,702 bp in size and have a GC content of 20.86%. The mitogenome contains two ribosomal RNA genes (*rnl* and *rns*), 26 tRNA genes, 15 conserved protein-coding genes (*atp6*, *atp8*, *atp9, nad1*, *nad2*, *nad3*, *nad4*, *nad4L*, *nad5*, *nad6, cox1*, *cox2*, *cox3, cob* and *rsp3*), seven free-standing ORFs (*orf102*, *orf188*, *orf257*, *orf269*, *orf277*, *orf283* and *orf412*), and one RNA subunit of the mitochondrial RNase P (*rnpB*). Only one intron, the type of Group IB, was detected in *cox1* gene. The result showed that seven PCGs genes (*atp6*, *atp9*, *cox1*, *cox2*, *nad2*, *nad3* and *nad*4) are on the forward strand, and other eight genes (*atp8*, *cox3*, *cob*, *nad1*, *nad4L*, *nad5*, *nad6* and *rps3*) are located on the reverse strand.

The mitochondrial genome sequences of 19 fungi were downloaded from GenBank for comparison (Figure 1). Two ascomycetes and two basidiomycetes were chosen as outgroups. Protein sequences from 14 PCGs were used for phylogenetic analyses. Amino acid sequences were aligned with MAFFT v7.051 (Katoh and Standley 2013) individually and concatenated with SequenceMatrix v1.7.8 (Vaidya et al. 2011). The best model of GTR + I+G for the maximum likelihood (ML) analysis was tested with Modeltest 3.7 (Posada and Crandall 1998). The phylogenetic tree was constructed using Maximum Likelihood (ML) method by RAxML 8.1.17 with 1,000 bootstrap replicates (Stamatakis 2014). In the clade of *Mortierellomycotina* Kerst. Hoffm., K. Voigt & P.M. Kirk 2011 (Figure 1), *Linnemannia amoeboidea* (W. Gams) Vandepol & Bonito 2020 is most closely related to *Podila verticillata* (Linnem.) Vandepol & Bonito 2020. Our results also confirm the close relationship of *Mortierellomycotina* Kerst. Hoffm., K. Voigt & P.M. Kirk 2011 to *Glomeromycotina* Spatafora & Stajich 2016 and *Mucoromycotina* Benny 2007 (Spatafora et al. 2016; Nie et al. 2019), and provide a further understanding of the phylogeny and evolution in basal fungi.

**Figure 1.**
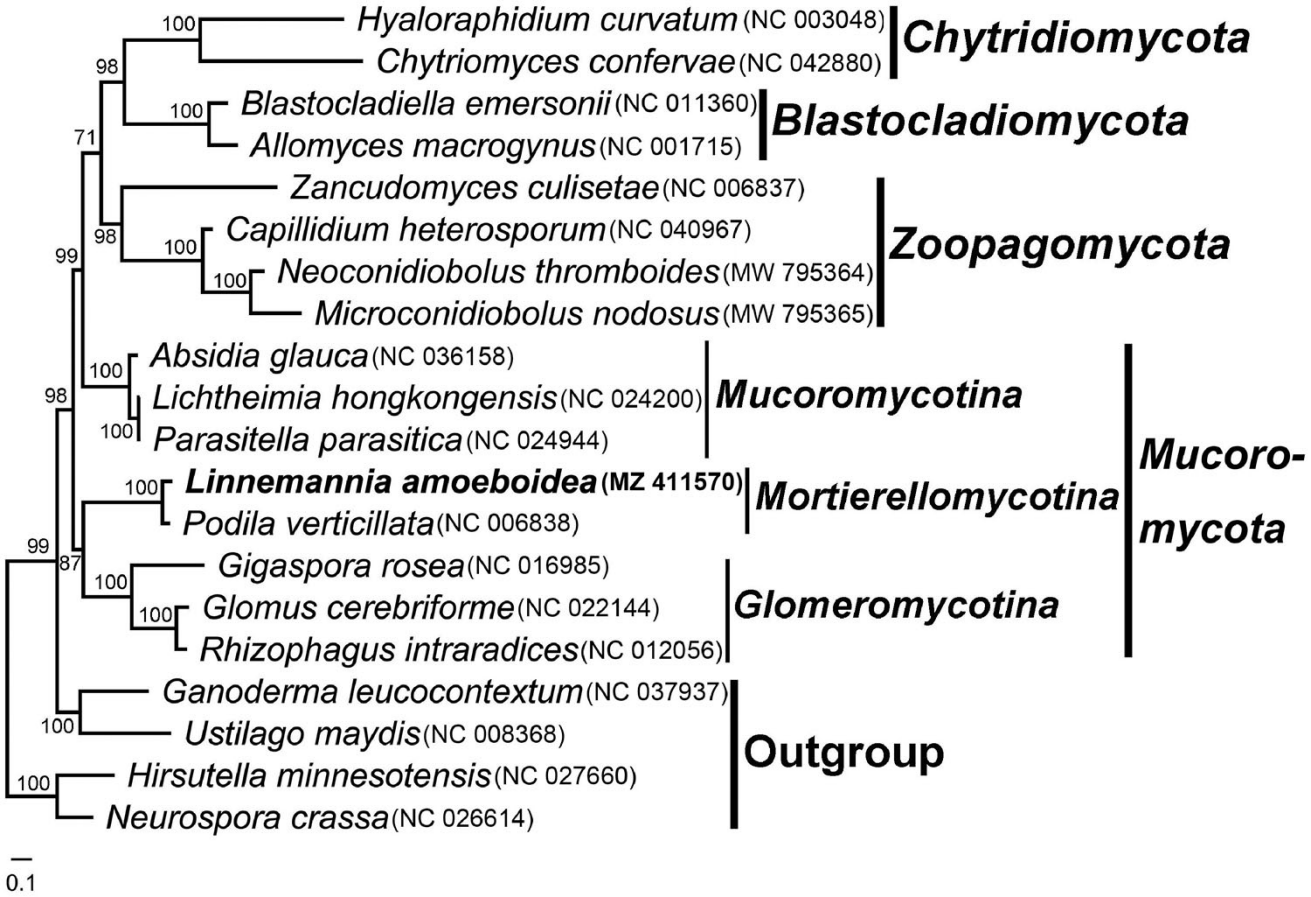
The phylogenetic tree constructed based on 14 mitochondrion encoded proteins. The 14 proteins included oxidase subunits (Cox1, 2, and 3), the apocytochrome b (Cob), ATP synthase subunits (Atp6, Atp8, and Atp9), NADH dehydrogenase subunits (Nad1, 2, 3, 4, 5, 6, and Nad4L). The following other 19 fungal mitogenomes were used in the phylogenetic analysis: Absidia glauca (Ellenberger et al. 2016), Allomyces macrogynus (Paquin and Lang 1996), Blastocladiella emersonii (Tambor et al. 2008), Capillidium heterosporum (Nie et al. 2019), Chytriomyces confervae (van de Vossenberg et al. 2018), Gigaspora rosea (Nadimi et al. 2012), Glomus cerebriforme (Beaudet et al. 2013), Hyaloraphidium curvatum (Forget et al. 2002), Lichtheimia hongkongensis (Leung et al. 2014), Microconidiobolus nodosus (Cai et al. 2021), Neoconidiobolus thromboides (Nie et al. 2021), Parasitella parasitica (Ellenberger et al. 2014), Podila verticillata (Seif et al. 2005), Rhizophagus intraradices (Lee and Young 2009), and Zancudomyces culisetae (Seif et al. 2005). Besides, Ganoderma leucocontextum (NC_037937), Hirsutella minnesotensis (Zhang et al. 2016), Neurospora crassa (NC_026614) and Ustilago maydis (NC_008368) were choosen as outgroups. Maximum likelihood bootstrap values (≥70 %) of each clade are indicated along branches. Scale bar indicates substitutions per site. The GenBank accession numbers are behind the Latin names.

## Data Availability

The genome sequence data that support the findings of this study are openly available in GenBank of NCBI at [https://www.ncbi.nlm.nih.gov] (https://www.ncbi.nlm.nih.gov/) under the accession no. MZ411570. The associated BioProject, SRA, and Bio-Sample numbers are PRJNA741872, SRP325947, and SAMN19911466 respectively.
